# Bedside rapid placement of nasointestinal feeding tube via ultrasound-guided stylet positioning in critical COVID-19 patients

**DOI:** 10.1186/s13054-020-02990-8

**Published:** 2020-06-18

**Authors:** Chuan-xi Chen, Zheng-deng Wei, Yong-jun Liu, Shou-zhen Cheng, Xiang-dong Guan

**Affiliations:** Department of Critical Care Medicine, The First Affiliated Hospital, Sun Yat-Sen University, Guangzhou, 510080 China

Dear Editor,

During our clinical work treating the breakout of coronavirus disease 2019 (COVID-19) in Wuhan, China. Good supportive care, including nutritional therapy, remains fundamental in managing critically ill patients with COVID-19, initiating enteral nutrition (EN) within the first 24–48 h after intensive care unit (ICU) admission in patients who are unable to eat unless contraindications exist [1]. Further, for those patients who were receiving mechanical ventilation or even in prone position, administration of analgosedation is known to slow down gastric emptying and thus post-pyloric feeding should be considered in case of persisting gastric retention [2, 3]. Due to the highly contagious nature of COVID-19, the implementation of heavy personal protective equipment (PPE) significantly increased the difficulties of performing clinical procedures, such as placing a bedside nasointestinal feeding tube without normal methods like auscultation with insufflation of air. To promptly and precisely finish the procedure with minimal exposure, we introduced the use of ultrasound-guided nasointestinal feeding tube positioning in those serious and critical patients [4].

In this report, we summarized our experience using ultrasound guidance for bedside nasointestinal feeding tube placement to significantly enhance the success rate while reducing time and labor.

We used a 140-cm-long nasointestinal tube with a stylet (20–9551, 10FR mm, CORPAK MedSystems Inc. IL, USA) and TE7 point of care ultrasound system (Mindray, Shenzhen, China). Ten minutes prior to the procedure, the patient was given metoclopramide 10 mg IV. Then, the tube was inserted intranasally for approximately 30–40 cm, and a linear transducer was placed on the neck to probe the tube image in the esophagus (Fig. 1a), to avoid misplacement into the trachea. After esophageal placement was confirmed, we advanced the tube to 50–60 cm into the stomach. Then, the patient was placed in a 45° right dorsal position; simultaneously, a convex transducer was used to position the antral sinus region. A quick shot of 10–20 ml normal saline was given to assure that the tube wall and stylet could be detected under scan. Tube entry was confirmed as observing the tram-track sign with stylet between the walls in the longitudinal section (Fig. 1b), and then tube was slowly pushed subsequently. Since using ultrasound is difficult to probe jejunal content, we introduced the “stylet withdraw and contrast” sign to position the tube, when the tube was inserted about 80–100 cm; the stylet was slowly withdrawn until the tip was detected in pylorus (Fig. 1c); and the length the stylet exposed at tail of the tube represented depth of the tip of tube beyond the pylorus. The normal length of duodenum is about 20–25 cm; therefore, when the stylet tip reached 20–25 cm post-pyloricly, it was considered as jejunal entry. And the procedure was concluded. Subsequently, the tip position was further confirmed by bedside abdominal radiology (Fig. 1d).

**Fig. 1:**
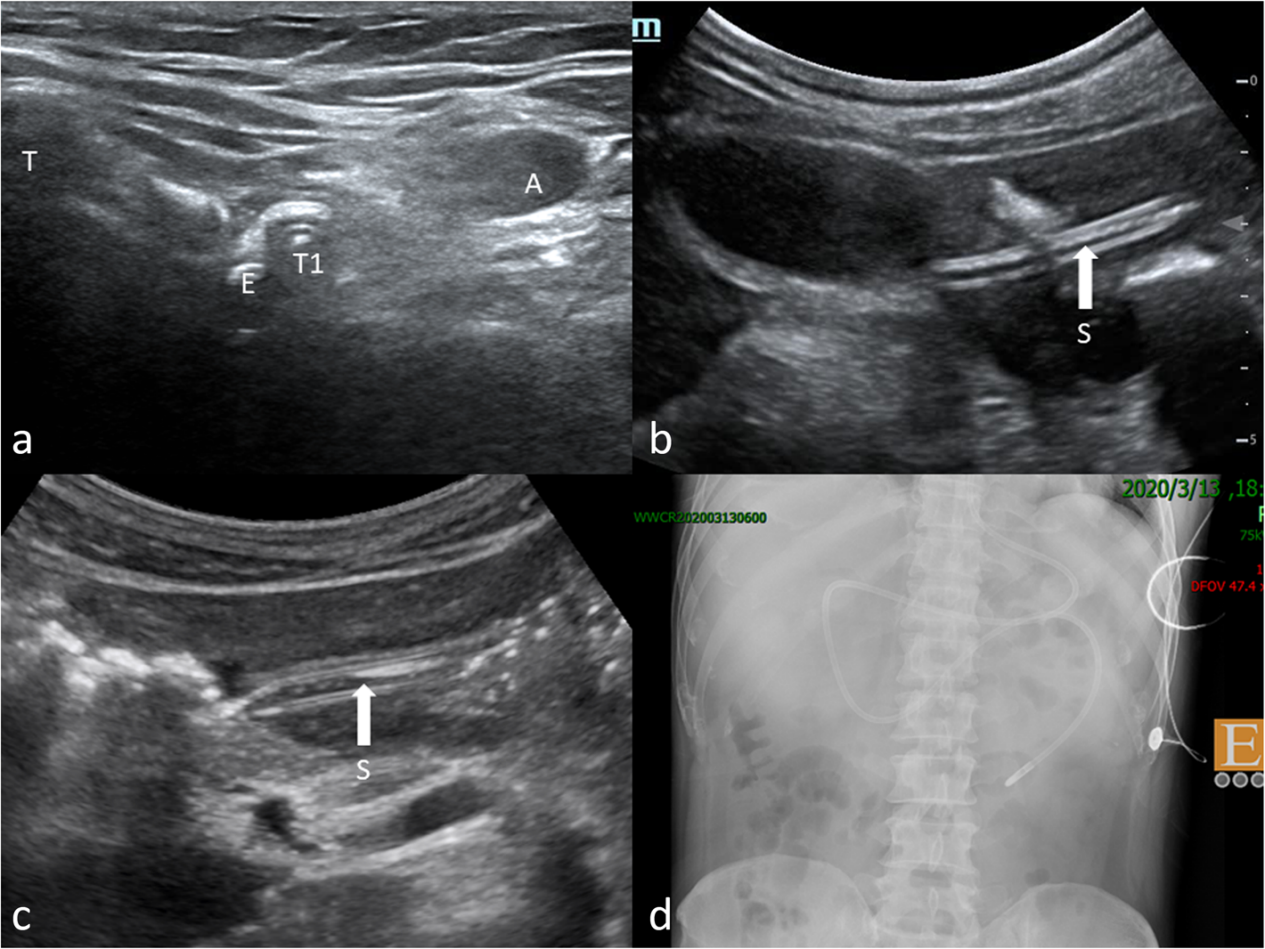
The location of the trachea (T), carotid artery (A), nasointestinal tube (T1), and esophagus (E) (**a**). Tram-track signs with stylet (S) in the longitudinal section (**b**). The stylet (S) tip in pylorus in the longitudinal section (**c**). Abdominal plain radiograph showed the tip of the tube (**d**)

Three patients received this ultrasound-guided nasointestinal feeding tube placement uneventfully in one attempt; the median time of procedure was 18 (15–25) min, which reduced procedural time and patients’ suffering and most importantly, minimized the exposure of medical staff in the high-virulent environment. Furthermore, the ultrasound-guided stylet method precisely helped us to confirm the tip position reaching the jejunum rather than the tube bending in stomach and maximally decreases aspiration risk, the accuracy of this novel method needs be tested on more cases. We propose to introduce this novel procedure to care for critical COVID-19 patients and protect medical staff.

## Data Availability

The data are available from the corresponding author.
